# Members of the class *Candidatus* Ordosarchaeia imply an alternative evolutionary scenario from methanogens to haloarchaea

**DOI:** 10.1093/ismejo/wrad033

**Published:** 2024-01-10

**Authors:** Dahe Zhao, Shengjie Zhang, Junyu Chen, Juanjuan Zhao, Peng An, Hua Xiang

**Affiliations:** State Key Laboratory of Microbial Resources, Institute of Microbiology, Chinese Academy of Sciences, Beijing 100101, China; State Key Laboratory of Microbial Resources, Institute of Microbiology, Chinese Academy of Sciences, Beijing 100101, China; College of Life Sciences, University of Chinese Academy of Sciences, Beijing 101408, China; State Key Laboratory of Microbial Resources, Institute of Microbiology, Chinese Academy of Sciences, Beijing 100101, China; State Key Laboratory of Microbial Resources, Institute of Microbiology, Chinese Academy of Sciences, Beijing 100101, China; State Key Laboratory of Microbial Resources, Institute of Microbiology, Chinese Academy of Sciences, Beijing 100101, China; College of Life Sciences, Sichuan Normal University, Sichuan 610068, China; State Key Laboratory of Microbial Resources, Institute of Microbiology, Chinese Academy of Sciences, Beijing 100101, China; College of Life Sciences, University of Chinese Academy of Sciences, Beijing 101408, China

**Keywords:** *Candidatus* Ordosarchaeia, evolutionary scenario, methanogen, haloarchaea, accelerated evolution, phylogenetic analysis

## Abstract

The origin of methanogenesis can be traced to the common ancestor of non-DPANN archaea, whereas haloarchaea (or *Halobacteria*) are believed to have evolved from a methanogenic ancestor through multiple evolutionary events. However, due to the accelerated evolution and compositional bias of proteins adapting to hypersaline habitats, *Halobacteria* exhibit substantial evolutionary divergence from methanogens, and the identification of the closest methanogen (either *Methanonatronarchaeia* or other taxa) to *Halobacteria* remains a subject of debate. Here, we obtained five metagenome-assembled genomes with high completeness from soda-saline lakes on the Ordos Plateau in Inner Mongolia, China, and we proposed the name *Candidatus* Ordosarchaeia for this novel class. Phylogenetic analyses revealed that *Ca.* Ordosarchaeia is firmly positioned near the median position between the *Methanonatronarchaeia* and *Halobacteria–Hikarchaeia* lineages. Functional predictions supported the transitional status of *Ca.* Ordosarchaeia with the metabolic potential of nonmethanogenic and aerobic chemoheterotrophy, as did remnants of the gene sequences of methylamine/dimethylamine/trimethylamine metabolism and coenzyme M biosynthesis. Based on the similarity of the methyl-coenzyme M reductase genes *mcrBGADC* in *Methanonatronarchaeia* with the phylogenetically distant methanogens, an alternative evolutionary scenario is proposed, in which *Methanonatronarchaeia*, *Ca.* Ordosarchaeia, *Ca.* Hikarchaeia, and *Halobacteria* share a common ancestor that initially lost *mcr* genes. However, certain members of *Methanonatronarchaeia* subsequently acquired *mcr* genes through horizontal gene transfer from distantly related methanogens. This hypothesis is supported by amalgamated likelihood estimation, phylogenetic analysis, and gene arrangement patterns. Altogether, *Ca.* Ordosarchaeia genomes clarify the sisterhood of *Methanonatronarchaeia* with *Halobacteria* and provide new insights into the evolution from methanogens to haloarchaea.

## Introduction

*Halobacteria* (also named haloarchaea or extreme halophiles) was one of the first identified archaeal lineages [1]. This class was initially affiliated with the phylum *Euryarchaeota* [2], and it was recently classified into a new phylum named *Halobacteriota* using a concatenated protein phylogeny in the Genome Taxonomy Database (GTDB) [3, 4]. *Halobacteria* are characterized by the ability to thrive in and depend on hypersaline environments [5, 6]. Most *Halobacteria* species require more than 1.5 M NaCl for growth [7]. The accumulation of sufficient inorganic salt (i.e. KCl) in the cytoplasm maintains an osmotic pressure equal to that in hypersaline environments [8]. Along with this thermodynamically favorable “salt-in” strategy, intracellular proteins accumulate a high proportion of acidic amino acids, leading to an acidified proteome [9, 10]. Moreover, these acidified proteins become dependent on high salt concentrations [10]. Although separated by a short phylogenetic distance from methanogens [11–13], *Halobacteria* species exhibit distinctive energy metabolism from that of methanogens. They cannot produce methane, and they generally exhibit aerobic respiration [14–16]. In fact, the methanogenic origin has been widely inferred to date back to the archaeal common ancestor [12, 17, 18]. The evolutionary pathway from anaerobic methanogens to aerobic and nonmethanogenic *Halobacteria* is an important topic.

Initially, *Halobacteria* were found to be closely related to *Methanomicrobia* [1], and their sisterhood was inferred in several phylogenetic analyses [2, 19, 20]. Later, *Methanonatronarchaeia* inhabiting hypersaline environments were reported as an alternative methanogenic sister [12], and some studies agree with this statement [21, 22]. However, the phylogenetic placement of *Methanonatronarchaeia* is also debated for long-branch attraction in the phylum *Halobacteriota* [23, 24]. The long branch of *Halobacteria* is possibly the result of fast adaptive evolution to (hyper)saline environments [25]. By removing the fast-evolving sites from the multiple sequence alignment prior to tree reconstruction, *Methanonatronarchaeia* was pushed to the root of the phylum *Halobacteriota* [23]. However, highly heterogeneous sites also contain phylogenetic information, and their removal might cause tree reconstruction artifacts [24]. Recently, the genomes of *Candidatus* Hikarchaeia species were assembled, and their inclusion as a transitional phase provided novel evidence that *Methanonatronarchaeia* were not the most closely related methanogens [13]. In the case that either *Methanomicrobia* or *Methanonatronarchaeia* represent the most closely related methanogens, *Halobacteria* were considered to have lost methanogenesis-related genes in the last common ancestor, and they laterally acquired many genes, including cytochrome *c* oxidase genes, from bacteria or other lineages [11, 13, 19, 21].

In this study, we obtained five highly complete *Candidatus* Ordosarchaeia genomes by assembling metagenomes collected from soda-saline lakes on the Ordos Plateau in China. Apart from analyzing their ecological distribution and metabolic potentials, our findings suggest that *Ca.* Ordosarchaeia diverged from an intermediate evolutionary position between the *Methanonatronarchaeia* and *Halobacteria–Hikarchaeia* lineages. The inclusion of *Ca.* Ordosarchaeia relieves the long-branch attraction of *Halobacteria* in phylogenetic analysis. Additional analyses based on their robust phylogeny provided new insights into the evolution from methanogens to haloarchaea.

## Materials and methods

### Sample collection, metagenome sequencing, and genome assembly

In total, 23 brine and sediment samples were collected from the ponds (salinity: 1%–33%) of soda-saline lakes (Habor Lake and Hutong Qagan Lake) on the Ordos Plateau of the Inner Mongolia Autonomous Region, China. Five deep sediment samples were used for enrichment cultivation. The sample site, physicochemical characterization, enrichment culture, DNA extraction, and metagenomic sequencing were described in a previous study [26–28]. Read quality control, contig assembly, and draft genome binning were performed with the same parameters as in previous research [27] based on the metaWRAP (v1.2.2) pipeline [29]. The representative genomes of *Ca.* Ordosarchaeia were included in the metagenome-assembled genomes (MAGs).

### Genomic estimation and taxonomic assignment

Genome size, contig number, N50, N90, maximum contig length, and G + C content were analyzed using the bbstats.sh script (25 July 2019) in the BBTools suite (sourceforge.net/projects/bbmap/). Genome completeness and contamination were determined using a lineage-specific workflow in CheckM (v1.1.3) [30]. Taxonomic classifications were assigned using the classify workflow in GTDB-Tk (v2.0.0) [31] based on Release 207 in GTDB. The genomes were annotated using Prokka (v1.14.6) with the settings of Archaea for annotation mode and RNAmmer (v1.2) for rRNA prediction [32, 33]. Aragorn (v1.2.41) was used to predict tRNAs [34], whereas Prodigal (v2.6.3) was used to predict coding genes [35]. The isoelectric point of each protein was predicted using the protein isoelectric point calculator IPC (January 2016) [36]. In total, 37 *Halobacteriota* genomes that were closely related to *Ca.* Ordosarchaeia were simultaneously analyzed as references (Supplementary Table S1). The average nucleotide identity between two genomes and the average amino acid identity (AAI) between two sets of predicted coding sequences from genomes were calculated using FastANI (v1.33) with default options [37] and the online AAI calculator (http://enve-omics.ce.gatech.edu/aai/index), respectively. The identities among the 16S rRNA gene sequences were estimated using BLASTN (v2.6.0+) [38].

### Phylogenetic analysis

The 3412 archaeal genomes in GTDB Release 207 were selected as a reference for phylogenomic analyses. Species trees were reconstructed from a concatenated set of 53 archaeal marker proteins [39], and the multiple sequence alignment file was produced during the classification workflow of GTDB-Tk [31]. The multiple sequence alignment could be directly used to rebuild the phylogenetic trees (named “untreated”), or it could be trimmed using BMGE v2.0 to remove regions with high entropy-like scores (weighted with the BLOSUM30 similarity matrix), which are not suited for phylogenetic inference [40]. Alternatively, ClipKIT (v1.3.0) was selected to identify and retain parsimony-informative sites [41]. Maximum-likelihood trees were reconstructed using IQ-TREE (multicore version 1.6.12) with an ultrafast bootstrap approximation based on 1000 replicates, and the best-fit model was chosen according to the Bayesian Information Criterion in ModelFinder [42]. To interpret the phylogenomic relationship, the multiple sequence alignment was subjected to the removal of sequences of *Ca.* Ordosarchaeia lineage and the removal of acidic amino acid residues by referencing the locations in different lineages. In addition, a Bayesian Markov chain Monte Carlo (MCMC)-based phylogenic analysis was performed using MrBayes (3.2.7a x86_64) [43, 44] with the following parameters: aamodelpr option for prior probability distribution, fixed gtr; rates for likelihood model, invgamma; number of rate categories for the gamma distribution, 4; number of runs, 2; number of generations, 3 000 000; number of chains for Metropolis-coupled MCMC variant, 4; samplefreq, 100; printfreq, 100; diagnfreq, 500; checkfreq, 1000; and fraction of samples that will be discarded in convergence diagnostics, 25%. Seventy-two archaeal genomes (including the 37 *Halobacteriota* genomes) were elaborately selected (listed in Supplementary Table S2) as a reference for the Bayes tree. A convergent Bayes tree was obtained when the average standard deviation of split frequencies was <0.01. The roots of all trees were set between DPANN and other phyla or between TACK and *Euryarchaeota* according to previous research [45]. All phylogenetic trees were visualized using the online tool Interactive Tree of Life (v6.6) [46].

### Functional annotation

The functions of encoding genes were predicted using an ortholog-based method. The five *Ca.* Ordosarchaeia genomes obtained in this research, along with 37 reference genomes, were used (Supplementary Table S1). All encoding genes were first functionally annotated according to the highest bit scores to the reference sequences in Archaeal Clusters of Orthologous Genes (arCOGs, the latest version ar18 at https://ftp.ncbi.nih.gov/pub/wolf/COGs/arCOG/) using BLASTP with threshold values of 30% identity and an evalue of 1e-5 [38]. Additionally, eggNOG-mapper (v2.0.0) was used to provide more information [47, 48], including Clusters of Orthologous Genes categories [49], Kyoto Encyclopedia of Genes and Genomes Orthology identifiers, and carbohydrate-active enzymes [50]. The provirus sequences in the genomes were predicted using VirSorter version 2.2.4 [51]. The defense systems were identified using DefenseFinder [52].

### Approximation of gene content and evolutionary events in ancestors

The presence or absence of each orthogroup containing four or more protein sequences and evolutionary events in ancestors was approximated across the archaeal domain according to our previous approach [27]. The 77 archaeal genomes, including 5 genomes obtained in this research, 37 representatives of *Halobacteriota*, and 35 genomes from other phyla, were selected. Briefly, the sequences of identical orthogroups were aligned using MAFFT (v7.407) with the L-INS-i method of high accuracy [53] and then trimmed using the heuristic automated1 method of trimAl (1.2rev59) [54]. Next, sequences containing too many gaps were excluded with the following options: minimum overlap of a position with other positions, 0.3; and minimum percentage of the satisfied positions, 50. A UFBOOT tree as a gene tree for each orthogroup was reconstructed using IQ-TREE with the settings (−m, LG + G; −bb, 1000; −wbtl) reported in previous research [55]. The frequencies of duplication, transfer (gene transfers from the lineage inside the species tree), loss, and origination (gene transfers from the lineages outside the species tree, or true gene originations), as well as the copy number of each orthogroup at each node, were inferred by maximum-likelihood estimation using the amalgamated likelihood estimation (ALE, v0.4) approach [56]. Considering the incompleteness of some genomes, the expected fraction of missing genes per genome was introduced from the completeness estimation of checkM. The orthogroups that exhibited a threshold of 0.3 in the raw reconciliation frequencies were counted [13].

### Phylogenetic analysis of marker proteins involved in methane metabolism

The methyl-coenzyme M reductase (Mcr) ABG subunits are marker proteins of anaerobic methane metabolism, including methanogenesis [19, 57, 58]. We selected the protein sequences of the McrABG subunits in *Methanonatronarchaeia* as seeds. The sequences of each subunit were screened using BLASTP with an evalue of 1e-3 from 3412 archaeal representative genomes in GTDB Release 207 for phylogenetic analysis. The putative alkyl-coenzyme M reductase (Acr) subunits were selected as outgroups for the phylogenetic trees according to previous research [18, 59]. The sequences were aligned using MAFFT with the L-INS-i method. After trimming using trimAl with the aforementioned settings, the maximum-likelihood trees were reconstructed using IQ-TREE with an ultrafast bootstrap approximation based on 1000 replicates, and the best-fit model was chosen.

### Relative abundance and geographic distribution

The relative abundance of genomes in the metagenomes was expressed as the reads per kilobase of contigs per million reads mapped calculated using CoverM (v.0.6.0) with the genome module (https://github.com/wwood/CoverM). The global distribution was based on the location of *Ca.* Ordosarchaeia detected from 16S rRNA gene and genomic sequences. Five *Ca.* Ordosarchaeia genomes were obtained from our samples, and one related genome (assembly accession: GCA_018609935.1) was collected from GTDB. The 16S rRNA gene sequences were screened from the nt database (update date, 16 October 2022) using BLASTN [38] against those retrieved from *Ca.* Ordosarchaeia genomes (two 16S rRNA sequences from the six *Ca.* Ordosarchaeia genomes). The taxonomic assignment was identified through phylogenetic placement. The 16S phylogenetic tree was reconstructed using IQ-TREE [42] after multiple sequence alignment and trimming using MAFFT [53] and trimAl [54], respectively. All options were set as previously described. The sequences that satisfied the following conditions were regarded as *Ca.* Ordosarchaeia: more than 500 bp in length; identity exceeding 82% with one of the reference sequences; and closer to reference sequences in the phylogenetic tree. The location information was retrieved from the metadata or by consulting the related publication, and the latitude and longitude values were mapped using the “maps” package (v3.4.0; https://CRAN.R-project.org/package=maps) in R project (v4.2.0).

## Results

### Biogeographic distribution and hypersaline adaptation of *Ca.* Ordosarchaeia, a novel class in the phylum *Halobacteriota*

**Figure 1:**
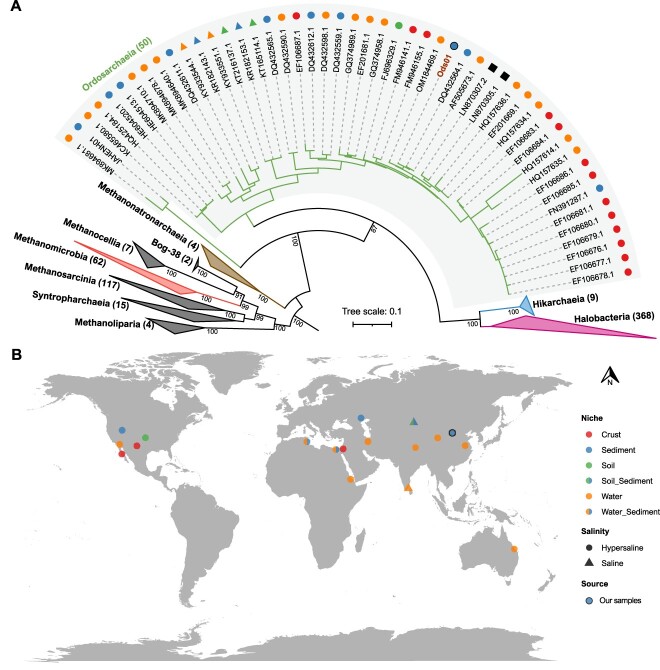
Phylogeny and biogeography of *Ca.* Ordosarchaeia; (A) phylogenetic diversity of *Ca.* Ordosarchaeia based on the 16S rRNA gene; the maximum-likelihood tree was reconstructed using the SYM + I + G4 model; the number at the node represents the percentage of ultrafast bootstrap support (>70%) after 1000 iterations; the number in the bracket behind the class name represents the number of sequences; Ods01 was obtained in this study; (B) geographic distribution of *Ca.* Ordosarchaeia; the latitude and longitude were mapped to illustrate the 16S rRNA genes more than 500 bp in length and the closely related phylogenetic relationships with *Ca.* Ordosarchaeia MAGs; colored triangles and circles denote environmental features, whereas black squares represent the sequences obtained from the host respiratory tract (Supplementary Table S4); “Hypersaline” indicates that the sample contains a high concentration of salts, whereas “saline” indicates that the sample contains salts, but the content was not provided; the blue circle with a black border marks the sequences obtained in this study.

Five MAGs with high completeness were obtained from soda-saline lakes on the Ordos Plateau of Inner Mongolia (Table 1). The MAGs were predicted to belong to the order JAHENH01 in the GTDB taxonomy database (Supplementary Table S1). However, they exhibited comparable average amino acid and 16S rRNA gene identities with *Halobacteria* (43%–52% and 79%–86%, respectively) as Marine Group IV archaea, which was recently renamed a novel class *Ca.* Hikarchaeia (47%–52% and 85%–89%, respectively; Supplementary Table S3). In addition, they shared lower identities (42%–45% and 80%–83%, respectively) with *Ca.* Hikarchaeia. Since they form a separate branch from *Halobacteria* and *Ca.* Hikarchaeia in phylogenetic analyses, we proposed that they be considered a novel class, which we named *Ca.* Ordosarchaeia (detailed in the Supplementary Information). We mainly utilized 16S rRNA gene sequences from *Ca.* Ordosarchaeia to explore their diversity and global distribution patterns (Supplementary Table S4). The majority of the 47 obtained sequences, which formed a cluster with Ods01, were found in various hypersaline or saline habitats, including brine, sediment, soil, and crust, whereas only two sequences (LN870305.1 and LN870307.2) were obtained from the human respiratory tract (Fig. 1A). Moreover, one sequence (MK894681.1) from hypersaline water exhibited high similarity to BinSanityLC-kmean-bin_57-bin_0-refined_5 (one MAG reported in the Bonneville Salt Flats [60]; renamed JAHENH01 in Fig. 1A). The geographic locations of environmental samples were mapped to illustrate the distribution. Samples were collected in Eurasia, Africa, America, and Australia (Fig. 1B). In summary, *Ca.* Ordosarchaeia exhibited a preference for hypersaline environments and a wide global distribution.

**Table 1:** Genomic features of the metagenome-assembled genomes (MAGs) affiliated with *Ca.* Ordosarchaeia in this study.

MAG	WGS accession number	Size (bp)	Contigs number	N50 (bp)	GC (mol%)	Completeness (%)	Contamination (%)	Reference for metagenome
Ods01	JAQZCZ000000000	1 865 478	133	25 316	60.82	97.06	2.64	[27]
Ods02	JAQZDA000000000	2 272 049	56	79 728	61.48	94.75	0.65	[27]
Ods05	JAQZDB000000000	1 224 804	315	4302	61.99	55.36	2.78	[27]
Ods03	JAQZDC000000000	1 686 625	416	5912	62.66	84.01	3.98	[27]
Ods04	JAQZDD000000000	1 703 903	411	5042	60.09	73.53	2.61	[26]

The five MAGs obtained in this study exhibited high abundance in soda-saline lakes or enrichment cultures with high salt concentrations (Supplementary Fig. S1A). Thus, these MAGs can be considered the representative genomes of *Ca.* Ordosarchaeia to decipher their hypersaline adaptation and ecophysiological potential. An acid shift in the isoelectric point profile of a proteome can be indicative of the salt-in strategy [28]. The predicted proteomes based on the genome sequences revealed that the five MAGs have acid-shifted isoelectric point profiles and possess similarly low average isoelectric points as *Haloferax volcanii* (Supplementary Fig. S1B and C), a model species of *Halobacteria* as a typical salt-in halophile [61]. In addition, the five *Ca.* Ordosarchaeia MAGs contained high proportions of acidic amino acid residues in the predicted proteomes (Supplementary Fig. S1D). Collectively, these findings suggest that *Ca.* Ordosarchaeia species employ the salt-in strategy to maintain sufficient osmotic pressure within the cytoplasm for hypersaline adaptation.

### *Methanonatronarchaeia* is the methanogen most closely related to the *Ordosarchaeia–Hikarchaeia–Halobacteria* branch

**Figure 2:**
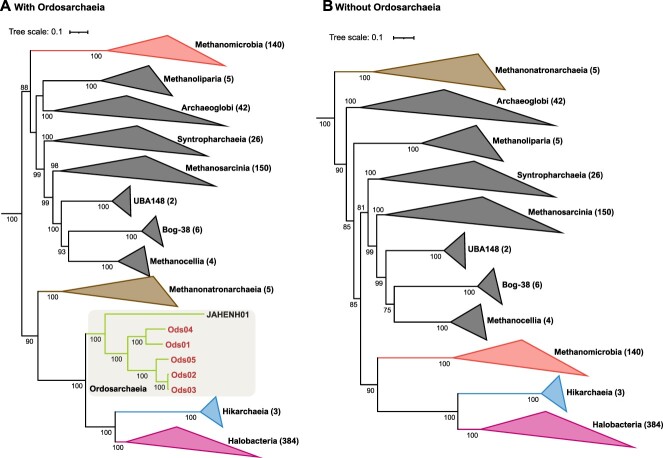
Phylogenomic analyses of the classes in the phylum *Halobacteriota*; maximum-likelihood trees (LG + F + G4 model) with (A) and without (B) *Ca.* Ordosarchaeia MAGs; the number at the node represents the percentage of ultrafast bootstrap support (>70%) after 1000 iterations; both phylogenomic trees are based on the 53 archaeal marker proteins, and the reference members are listed in Supplementary Table S2; some branches are collapsed, and the number in the bracket represents the number of genomes.

The phylogenetic tree based on 16S rRNA genes revealed that *Ca.* Ordosarchaeia is a deep lineage of *Ca.* Hikarchaeia and *Halobacteria*. Moreover, these three classes form a sister branch with *Methanonatronarchaeia* (Fig. 1A). To determine the placements of these four classes and their evolutionary relationships, we conducted whole genome-based phylogenetic analyses. The phylogenomic tree based on the 53 “top-ranked” archaeal marker proteins indicated that the four classes form a distinct clade from the other classes within the phylum *Halobacteriota* with 100% bootstrap support, and *Methanonatronarchaeia* was located at the root of the clade with 90% bootstrap support (Fig. 2A). To mitigate potential errors caused by long-branch attraction in the phylogeny of *Halobacteriota*, we employed multiple approaches for tree reconstruction. First, we removed the fast-evolving sites in the multiple sequence alignment prior to maximum-likelihood tree reconstruction using two different tools, namely, BMGE and ClipKit [40, 41]. In this analysis, the phylogenetic positions of the four classes remained unchanged (Supplementary Fig. S2). Second, we generated a consensus tree using Bayesian inference, which positioned the four classes with 100% probability for each partition, similar to the maximum-likelihood trees (Supplementary Fig. S3). Third, we performed a maximum-likelihood analysis excluding the *Ca.* Ordosarchaeia lineage, which resulted in a significant alteration of the tree structure. In this scenario, *Methanonatronarchaeia* was relocated to the root of the phylum *Halobacteriota* with 100% bootstrap support (Fig. 2B). These modifications collectively indicate that incorporating *Ca.* Ordosarchaeia at an intermediate position between *Methanonatronarchaeia* and *Halobacteria* substantially enhances the robustness of phylogenetic analyses.

To identify the factors influencing the phylogenetic analysis of *Halobacteriota*, we conducted artificial treatments to remove the acidic amino acid residues, i.e. glutamate and aspartate, in the multiple sequence alignment based on the 53 archaeal marker proteins before the reconstruction of maximum-likelihood trees. *Methanonatronarchaeia* was also pushed down to the root of the whole phylum by referencing the locations of the sites in each representative sequence from *Halobacteria*, *Ca.* Hikarchaeia, *Ca.* Ordosarchaeia, and *Methanonatronarchaeia* (Supplementary Fig. S4A–D). However, when considering *Methanomicrobia* and *Methanosarcinia*, the position of *Methanonatronarchaeia* close to *Ordosarchaeia–Hikarchaeia–Halobacteria* remained unchanged (Supplementary Fig. S4E and F). We observed that the most selected tree models in ModelFinder were LG + G4, but the model became LG + F + G4 when referring to the *Ca.* Hikarchaeia sequence. Therefore, we used the LG + G4 model for the treatment based on *Ca.* Hikarchaeia (Supplementary Fig. S4G). Both models exhibited the same topological structure at the class level. Collectively, the trees based on the different treatments suggest that the clade of *Halobacteria*, *Ca.* Hikarchaeia, *Ca.* Ordosarchaeia, and *Methanonatronarchaeia* share conserved acidic amino acid residues at certain sites that probably drive the evolution of this clade. In addition, the acidic amino acid residues accumulated in the early evolutionary phase might not have been driven solely by hypersaline conditions considering the slightly halophilic *Ca.* Hikarchaeia and MAG BinSanityLC-kmean-bin_57-bin_0-refined_5 (discussed below).

### Energy metabolism comparison supports *Ca.* Ordosarchaeia as an intermediate phase between *Methanonatronarchaeia* and *Halobacteria*

**Figure 3:**
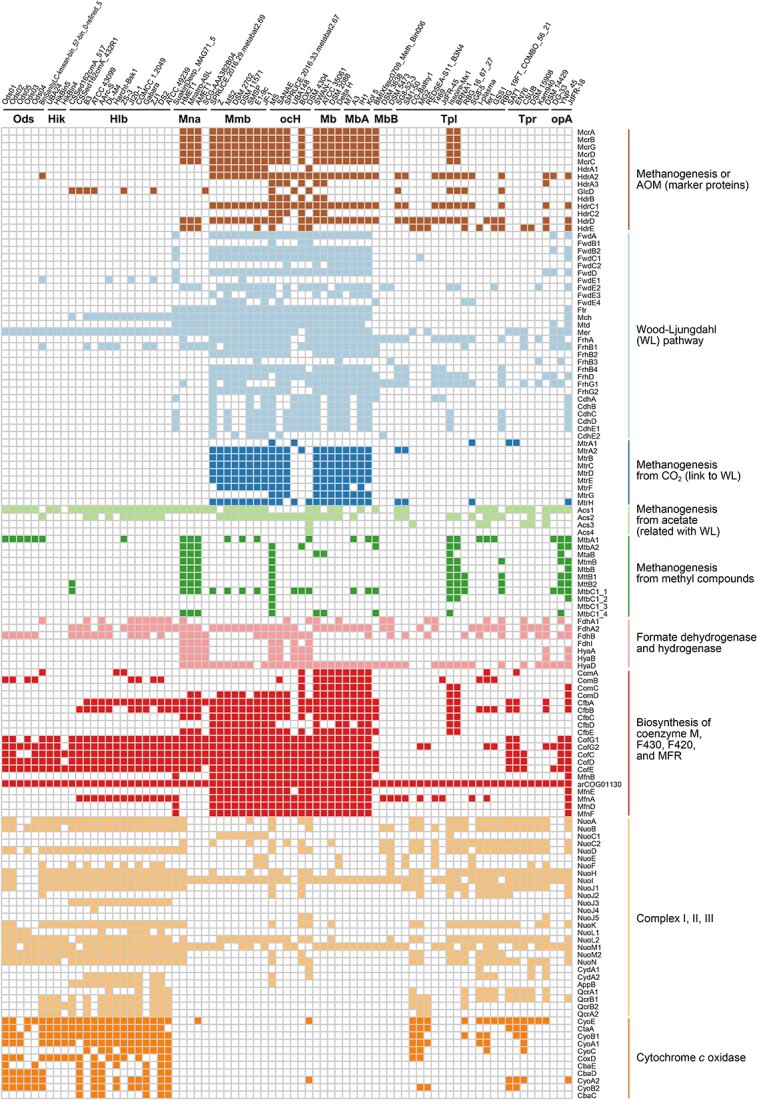
Energy-related metabolism of *Ca.* Ordosarchaeia compared with reference lineages; the colored and white squares denote the presence and absence of functional genes in the genomes, respectively, and the full names of the genes and their functions are listed in Supplementary Table S6; the assembly accession numbers of the genomes are presented in Supplementary Table S1 and Supplementary Fig. S5; the abbreviations of superordinate taxonomies were as follows: Ods, *Ca.* Ordosarchaeia; Hik, *Ca.* Hikarchaeia; Hlb, *Halobacteria*; Mna, *Methanonatronarchaeia*; Mmb, *Methanomicrobia*; ocH, other classes in the phylum *Halobacteriota*; Mb, *Methanobacteriota*; MbA, *Methanobacteriota*_A; MbB, *Methanobacteriota*_B; Tpl, *Thermoplasmatota*; Tpr, *Thermoproteota*; opA, other phyla in archaea.

The main difference between methanogens and haloarchaea is energy metabolism. *Ca.* Ordosarchaeia MAGs do not have any *mcr* genes, but they contain cytochrome *c* oxidase genes (Fig. 3). Some members (Ods01 and Ods04) even encode bacteriorhodopsin and the related protein arCOG02947 (Supplementary Fig. S5, Supplementary Tables S5 and S6). Obviously, their metabolism based on functional gene prediction was similar to that of *Halobacteria* even though they do not harbor cytochrome *b* and cytochrome *bd*-type quinol oxidase for cytochrome *c* reductase (Fig. 3). The electron transfer chain is possibly incomplete in *Ca.* Ordosarchaeia, or electron transfer from quinol to cytochrome *c* might be accomplished by different enzymes or via a different pathway.

*Methanonatronarchaeia* members were characterized to perform formate- or H_2_-dependent methyl-reduction to produce methane [12, 62]. We annotated the related genes, including formate dehydrogenases, [NiFe]-hydrogenase, multiple methyltransferases, and Mcr and CoB-CoM heterodisulfide reductase, in some *Methanonatronarchaeia* genomes (Fig. 3). However, we did not find the complete pathway in any *Ca.* Ordosarchaeia MAG, but rather, we only identified some subunits, such as methylcobalamin:coenzyme M methyltransferase subunit A (MtbA) in methylamine/dimethylamine/trimethylamine metabolism and Fe-S-cluster-containing formate dehydrogenase (FdhB) for formate utilization. Hydrotroilite (FeS × nH_2_O) has been reported to be necessary for both the growth and methanogenic activity of *Methanonatronarchaeia*, although whether hydrotroilite affects the activity of FdhB is unclear [12]. In contrast, most *Methanomicrobia* species do not carry the genes to reduce methylated compounds or *fdhB* (Fig. 3). Although the metabolic processes *of Ca.* Ordosarchaeia remain unidentified, the presence of *mtbA* and *fdhB* in *Ca.* Ordosarchaeia supports its closer relationship with *Methanonatronarchaeia* than with *Methanomicrobia*. In addition, the absence of *uvrABC* genes involved in nucleotide excision repair in both *Ca.* Ordosarchaeia and *Methanonatronarchaeia* is consistent with their close relationship. In comparison, these genes are widely distributed among *Halobacteria*, *Ca.* Hikarchaeia, *Methanomicrobia*, and other classes within the phylum *Halobacteriota* (see Supplementary Information).

Moreover, the five MAGs of *Ca.* Ordosarchaeia contain genes involved in many types of ion transporters, including the Kef- and Trk-type K^+^ transport systems and Mnh Na^+^/H^+^ antiporters (Supplementary Fig. S5, Supplementary Table S6A). Ion transport performed by salt-in halophiles plays an important role in ion homeostasis and osmotic pressure balance [63]. The *Ca.* Ordosarchaeia MAGs also featured high copy numbers of universal stress protein A (UspA), especially arCOG02053 and arCOG00449 (Supplementary Table S6A), a marker protein of adaptation to extreme (e.g. hypersaline) environments [12]. The presence of these genes further supports the hypothesis of the salt-in strategy. Additionally, we found *that Ca.* Ordosarchaeia MAGs harbored genes related to central carbohydrate metabolism, oxidative phosphorylation, terpenoid biosynthesis, and DNA repair but lacked the potential for carbon fixation (Supplementary Fig. S5). Therefore, it is likely that *Ca.* Ordosarchaeia species are chemoheterotrophs with an aerobic respiration-based lifestyle.

### *Methanonatronarchaeia* possesses an evolutionarily distant Mcr across the robust phylogeny of archaea, including *Ca.* Ordosarchaeia

**Figure 4:**
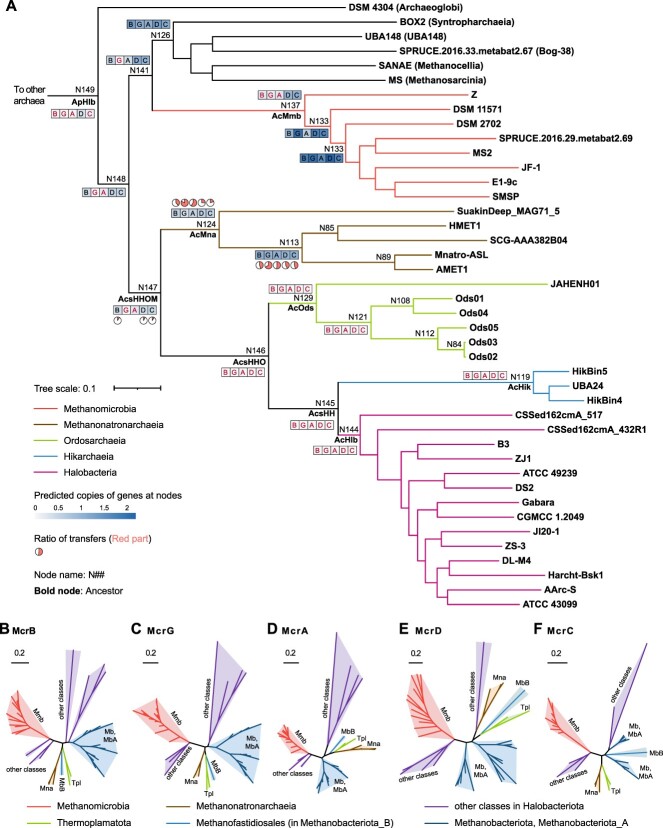
Ancestral inference and phylogenetic analyses of marker proteins involved in methane metabolism; (A)inference of the marker proteins McrBGADC (abbreviated as BGADC for the five proteins) involved in methane metabolism in the main ancestors across the archaea domain; the symbols BGADC are colored to indicate that the copies of the encoding genes are calculated as <0.3; the ancestral potential was estimated by reconciling UFBOOT gene trees against an archaeal species tree using ALE; the ratios of transfers were calculated by dividing the frequencies of transfers by the frequencies of copies; the maximum-likelihood tree (LG + F + I + G4 model) of the consensus species was reconstructed using ultrafast bootstrap approximation based on the 53 archaeal marker proteins from 77 representative genomes (listed in Supplementary Table S2), and the node numbers are presented in Supplementary Fig. S6; the colored and black branches mark the different classes in the phylum *Halobacteriota*, and the other phyla are not presented; (B–F) phylogenies of five marker proteins from 77 representative genomes; the colored branches mark the different taxonomic sources of the proteins in the archaea.

**Figure 5:**
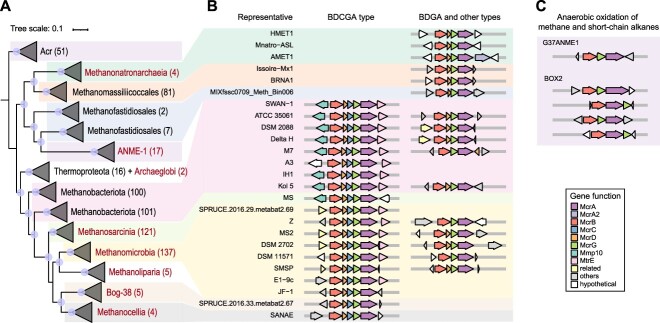
Arrangement of *mcr* genes across the phylogeny of subunit A; (A) phylogenetic analysis of the McrA subunit and its homologs; the maximum-likelihood tree was reconstructed using the LG + F + I + G4 model; the circle at the node signifies >70% ultrafast bootstrap support based on 1000 iterations; some branches are collapsed, and the number in the bracket represents the number of sequences; the label is colored to indicate the sequences that were affiliated with lineages of the phylum *Halobacteriota*; all sequences are listed in Supplementary Table S7; the detailed approaches are described in the Methods; (B) organization comparison of representative *mcr* gene clusters involved in methanogenesis; (C) organizational comparison of representative *mcr* gene clusters involved in the anaerobic oxidation of methane and short-chain alkanes; the direction and color of each arrow denote the gene direction and function, respectively; the contig ID and position of each gene cluster are listed in Supplementary Table S8.

During archaeal evolution, *Halobacteria* are considered to have lost methanogenic capability from their closely related methanogenic ancestor [11–13]. The trees of relevant genes were reconciled against the robust archaeal species tree in this study to reconstruct the ancestral states using the state-of-the-art ALE approach. Orthogroups that exhibited a threshold of 0.3 in the raw reconciliation frequencies were selected to avoid missing true events [13]. In our evolutionary approximation, the ancestor of *Halobacteria*, *Ca.* Hikarchaeia, *Ca.* Ordosarchaeia, and *Methanonatronarchaeia* (AcsHHOM for short at node N147) might not have harbored all of the genes involved in methanogenesis because the frequencies of the Mcr genes *mcrGA* are predicted to be no more than 0.3 (Fig. 4A). The frequencies of the *mcrBGA* genes in the common ancestor of *Methanonatronarchaeia* (node N124) were greater than 0.3 but much lower than 1.0, whereas they reached or approached 1.0 in the later ancestor (node N113). Approximately 40% of the *mcrB* genes and more than half of the *mcrGA* genes of *Methanonatronarchaeia* were estimated to be obtained at nodes N124 and N113 through horizontal gene transfer (HGT) (Fig. 4A). Similarly, a significant proportion of *mcrDC* genes in *Methanonatronarchaeia* appeared to have been laterally acquired at node N113. According to the principle of ALE analysis, the *mcr* genes harbored by partial *Methanonatronarchaeia* members might be separated by a longer phylogenetic distance from the genes of most methanogenic lineages in the phylum *Halobacteriota* compared to those outside the phylum. This was supported by phylogenetic analyses of the sequences of the five Mcr proteins (Fig. 4B–F). Taken together, these results suggest that the *mcr* genes were likely acquired laterally by the common ancestor (node N124) or a descendant ancestor (node N113) of *Methanonatronarchaeia* (Supplementary Table S6B, Supplementary Fig. S6). Considering the widespread distribution of methane metabolism in archaea [19, 57, 64], we collected the amino acid sequences of the representative *mcrBGA* genes across the whole archaeal domain and then reconstructed their phylogenies to trace the source of these genes. In the maximum-likelihood tree based on McrA (also including the homolog alkyl-coenzyme M reductase AcrA subunit) sequences, *Methanomicrobia*, *Methanosarcinia*, *Methanoliparia*, Bog-38, and *Methanocellia* clustered together, whereas other sequences were located outside this cluster with high confidence (greater than 70% bootstrap support). In other words, *Methanonatronarchaeia* and ANME-1 are closer to *Methanomassiliicoccales* and *Methanofastidiosales*, and *Archaeoglobi* is in the branch with *Thermoproteota* (Fig. 5A). The phylogenetic trees based on McrB- and McrG-like sequences featured similar topological structures (Supplementary Fig. S7A and B).

Furthermore, we compared the gene organization within the *mcr* cluster. Each *Methanonatronarchaeia* genome carried a single *mcrBDGA* gene cluster organized in a head-to-tail manner (Fig. 5B). This gene arrangement, termed the BDGA type in this study, is identical to those in *Methanomassiliicoccales* and *Methanofastidiosales*, the McrABG sequences of which share high similarity. However, it differs from the gene arrangement observed in *Methanosarcinia*, *Methanomicrobia*, *Methanoliparia*, Bog-38, and *Methanocellia*, which belong to the same phylum as *Methanonatronarchaeia*, but their representative genomes consistently contained a BDCGA-type *mcr* cluster. Additionally, in *Methanomicrobia*, approximately half of the representative genomes carried an additional BDGA-type cluster, whereas some genomes featured other types (Fig. 5B). Unexpectedly, the multiple *mcr* clusters of the two lineages shape separate branches in the phylogenetic trees of McrA, McrB, and McrG (Fig. 5A, Supplementary Fig. S7). In other words, McrABGs located in different types in the same taxonomic class might have been generated through gene duplication. Furthermore, certain genomes within the phylum *Methanobacteriota* also exhibited two different types of *mcr* clusters (Fig. 5B and C). Considering the expansion and conservation of the BDCGA type in *Halobacteriota* and *Methanobacteriota* (excluding *Methanonatronarchaeia* and *Methanofastidiosales*, respectively), we hypothesize that this type was vertically inherited in both phyla and that other types were later derived from it (in the lineages possessing two or more types of *mcr* gene clusters), or that all of them were lost in lineages lacking *mcr* genes. With respect to the evolutionarily distant BDGA types in *Methanonatronarchaeia*, *Methanofastidiosales*, and *Methanomassiliicoccales*, they might have diverged in certain methanogens in the early phase and later horizontally transferred to other lineages (such as *Methanonatronarchaeia*). Furthermore, considering *Methanonatronarchaeia* as the sole methanogenic class in the branch of *Methanonatronarchaeia*, *Ca.* Ordosarchaeia, *Ca.* Hikarchaeia, and *Halobacteria*, the hypothesis of HGT is reasonable. In fact, another hypothesis involving the horizontal transfer of these genes is that the *mcr* genes were ancestrally present in the *Methanonatronarchaeia* lineage and that the horizontally transferred *mcr* genes replaced the ancestral genes. To evaluate this possibility, we reconstructed the phylogenetic tree of CoB-CoM heterodisulfide reductase subunits D and E (HdrDE) while considering the absence of coenzyme M biosynthesis in *Methanonatronarchaeia* and the limited distribution of MtbA in other methanogens (Fig. 3). Some *Methanonatronarchaeia* members were found to possess two copies of *hdrD* and *hdrE*, and one copy was found to form a gene cluster with *mcrC* (Supplementary Fig. S8A). In the phylogeny of HdrD, *Thermoplasmatota* exhibited the highest diversity of proteins, and the clustered HdrD of *Methanonatronarchaeia*, along with certain sequences in *Methanomicrobia* and other phyla, was adjacent to *Thermoplasmatota* but distant from the main branch of *Methanomicrobia* and other classes in the phylum *Halobacteriota* (Supplementary Fig. S8B). A similar condition was observed for another copy. However, there were insufficient HdrE sequences for the relevant analysis (Supplementary Fig. S8C). These results indicate that HdrD, McrC, and HdrE in *Methanonatronarchaeia* were also acquired through HGT, thereby refuting the hypothesis of orthologous displacement. In summary, we propose an alternative course of haloarchaeal evolution based on the divergent *mcr* gene cluster in *Methanonatronarchaeia* and the robust phylogenetic relationship of *Methanonatronarchaeia* and *Ordosarchaeia–Hikarchaeia–Halobacteria*.

## Discussion

*Ca.* Ordosarchaeia is widely distributed in hypersaline habitats. The presence of acid-shifted proteomes and multiple copies of *uspA* genes in *Ca.* Ordosarchaeia species support their adaptation to hypersaline environments, although the occurrence of certain clones in the nonsaline respiratory tract appears to challenge this claim. *Halobacteria* species are well known for their ability to thrive in hypersaline environments [7, 65]. Nevertheless, with the development of high-throughput sequencing, certain *Halobacteria* species have also been discovered in the human gut, representing a nonsaline environment [66, 67]. Several cultivated *Halobacteria* strains, e.g. *Halalkalicoccus*, *Haladaptatus*, *Halocatena*, and *Halomarina oriensis* (Supplementary Table S9), can maintain cellular integrity even under nonsaline conditions. Importantly, these exceptions do not nullify the hypersaline characteristics of *Halobacteria*. Similarly, it is reasonable to assess the extreme halophilic features of *Ca.* Ordosarchaeia. However, it remains difficult to determine the mechanism by which these *Halobacteria* and *Ca.* Ordosarchaeia species reconcile the salt-in strategy and their dependence on high salt-dependent enzymes with nonsaline conditions or assess whether they enter a dormant state under such conditions.

The long-branch attraction in the phylogenetic analysis of *Halobacteriota* cannot be overlooked because the phylogenetic relationship between *Halobacteria* and *Methanonatronarchaeia* is equivocal [12, 13, 23, 24]. Facing the same problem, the phylogeny of *Candidatus* Nanohaloarchaeota has also been a subject of debate, with some studies placing them within the phylum *Halobacteriota* [25, 68]. This potential phylogenetic artifact might arise from the rapid evolution of *Halobacteria* and *Ca.* Nanohaloarchaeota in response to saline adaptation. The discovery of *Ca.* Ordosarchaeia provides valuable insight, as it is placed at a central position between *Methanonatronarchaeia* and *Halobacteria* in the phylogenies, and the inclusion of *Ca.* Ordosarchaeia helps to break the long distance between *Halobacteria* and *Ca.* Hikarchaeia and other lineages. Additionally, *Methanonatronarchaeia* consistently moved close to *Halobacteria*, *Ca.* Hikarchaeia, and *Ca.* Ordosarchaeia from the root. A recent report also supports that improved taxon sampling is crucial for obtaining robust phylogenies [69]. Apparently, when the intermediate lineage has not been discovered, it will be an effective approach to select unambiguously aligned sites (same as removing highly variable sites) to estimate phylogenetic relationships in many cases [70–72], but this might not be suitable for *Halobacteria*. We agree with the view that fast-evolving sites contain evolutionary signals and that their removal can sometimes lead to phylogenetic artifacts [24]. *Ca.* Hikarchaeia was located at the transitional stage, but the placement of *Methanonatronarchaeia* at the root appears to be accurate [13]. In our opinion, *Ca.* Hikarchaeia is located excessively close to *Halobacteria* but far from *Methanonatronarchaeia*. Therefore, including *Ca.* Hikarchaeia does not clarify the overall phylogeny.

Fast adaptive evolution to saline environments was considered the cause of the long-branch formation of *Halobacteria* [25]. In combination with the accumulation of acidic amino acids in the proteomes of salt-in lineages for hypersaline survival, the results of phylogenetic analyses after removing glutamate and aspartate appear to support hypersaline selection. *Ca.* Hikarchaeia species might be slight halophiles, whereas they also feature certain acidic amino acid sites similar to other extreme halophilic organisms, such as *Halobacteria*, *Methanonatronarchaeia*, and *Ca.* Ordosarchaeia species. Marine microorganisms generally produce or import organic osmolytes, e.g. ectoine, glycine betaine, *N*^ε^-acetyl-β-lysine, and β-glutamine, to cope with high-salinity environments [73–76]. Based on these observations, we hypothesize that the last common ancestor of *Methanonatronarchaeia*, *Ca.* Ordosarchaeia, *Ca.* Hikarchaeia, and *Halobacteria* is a slight salt-in halophile. The slight salt-in strategy could be identified by measuring the intracellular salt concentration in a pure culture of *Ca.* Hikarchaeia or by providing biochemical evidence to test the optimal salinity of intracellular enzymes in future studies. In fact, the salt-in strategy might have independently originated in different archaeal classes, such as the halophilic lineage *Candidatus* Halarchaeoplasmatales in the phylum *Thermoplasmatota* [28] and nanosized *Ca.* Nanohaloarchaeota [27, 77]. Similarly, extreme salt-in adaptation might have independently evolved in *Halobacteria*, *Ca.* Ordosarchaeia, and *Methanonatronarchaeia* from the slight salt-in strategy of their common ancestor. Apparently, we cannot dismiss the possibility that the common ancestor was an extreme halophile and that the classes later returned to slightly halophilic lineages.

Generally, the inference of the evolutionary process of archaeal methane metabolism relies on a consensus of phylogenetic analyses conducted with each methanogenic lineage [12, 18, 19, 59, 64]. Possibly because methanogenesis was commonly inferred to originate in the common ancestor of non-DPANN Archaea [12, 18, 57, 59, 64, 78, 79], *Halobacteria* were thought to have lost methanogenesis from the last common ancestor with the most closely related methanogen, and this ancestor was previously considered to harbor methanogenesis-related genes regardless of which lineage was the most closely related methanogen [12, 13]. Based on the robust phylogeny of *Methanonatronarchaeia* and *Halobacteria* in this study, we propose an alternative possibility that *Methanonatronarchaeia* might have laterally acquired *mcrABGDC* and *hdrDE* genes from *Methanomassiliicoccales* or *Methanofastidiosales*, considering the phylogenetically and organizationally divergent *mcr* gene cluster of *Methanonatronarchaeia* from closely related methanogens and the absence of *mcr* genes in *Halobacteria*, *Ca.* Hikarchaeia, *Ca.* Ordosarchaeia, and some *Methanonatronarchaeia* members. Inferring horizontal transfer of methanogenesis genes was reasonable because similar events were predicted to occur in other methanogens, e.g. *Methanomassiliicoccales* [18, 80]. Although *Methanonatronarchaeia* was generally found in hypersaline environments with neutral or alkaline pH [12, 62, 81], we believe that the lateral transfer of *mcrABG* can occur in extreme environments. In fact, most *Methanomassiliicoccales* species were reported in host-associated or nonhypersaline natural environments [82, 83], but some members are certain to be detected in hypersaline and alkaline environments as the source of HGT [26, 28, 84]. Similarly, *Methanofastidiosales* species were also found in soda lake sediment [85].

This study presented the novel class *Ca.* Ordosarchaeia within the phylum *Halobacteriota* and characterized its widespread distribution in hypersaline environments, as well as its adaptation strategy and metabolic potential based on its MAGs. Additionally, the inclusion of *Ca.* Ordosarchaeia significantly reduced the phylogenetic distance of *Halobacteria* and *Ca.* Hikarchaeia from the most closely related lineages and apparently relieves the problem of long-branch attraction in phylogenetic analyses. Based on the phylogenetic consensus and the divergent *mcr* gene cluster for methanogenesis found in the *Methanonatronarchaeia*-related branch, an alternative evolutionary scenario from methanogens to haloarchaea was proposed. Specifically, the ancestral lineage of *Halobacteria* and the closely related methanogen *Methanonatronarchaeia* underwent an initial loss of *mcr* genes, followed by the later acquisition of *mcr* from phylogenetically distant methanogens.

## Author contributions

Hua Xiang and Dahe Zhao conceived the study. Shengjie Zhang and Dahe Zhao carried out the metagenomic analysis and genomic binning. Dahe Zhao performed the bioinformatic analyses on phylogenies, comparative genomics, functional annotation, evolutionary event approximation, biogeography, and statistics. Dahe Zhao prepared the figures and drafted the manuscript. Shengjie Zhang, Junyu Chen, Juanjuan Zhao, Peng An, and Hua Xiang participated in the functional interpretation, discussion and manuscript revision. All authors read and approved the final manuscript.

## Conflicts of interest

The authors declare no conflict of interest.

## Funding

This study was funded by the National Natural Science Foundation of China (No. 92251302 and 32000046), and supported by Central Asian Drug Discovery and Development Center of Chinese Academy of Sciences (No. CAM202202).

## Data availability

The five *Ca.* Ordosarchaeia genomes are available from the NCBI under the BioProject identifier PRJNA924292. Metagenomic sequencing data have been deposited in BioProject identifiers PRJNA549802, PRJNA679647, and PRJNA769545. Raw data (including protein sequence, functional annotation, protein cluster, multiple sequences alignment, amalgamated likelihood estimation for history approximation, and scripts, etc.) generated in this study are available through [https://doi.org/10.6084/m9.figshare.21989720].

## Supplementary Material

Table_S1_wrad033

Table_S2_wrad033

Table_S3_wrad033

Table_S4_wrad033

Table_S5_wrad033

Table_S6_wrad033

Table_S7_wrad033

Table_S8_wrad033

Supplementary_Information_wrad033
