# Supplementary material for: Members of the class Candidatus Ordosarchaeia imply an alternative evolutionary scenario from methanogens to haloarchaea
Source: ISME J. 2024 Jan 10;18(1):wrad033. doi: 10.1093/ismejo/wrad033 (PMC10873845; doi:10.1093/ismejo/wrad033)
Supplement: Supplementary_Information_wrad033 [file supplementary_information_wrad033.pdf]

Supplementary Information

Members of the class *Candidatus* Ordosarchaeia imply an alternative evolutionary scenario from methanogens to haloarchaea

Dahe Zhao[1]*, Shengjie Zhang[1,2], Junyu Chen[1], Juanjuan Zhao[1], Peng An[1,3], Hua Xiang[1,2]*

1 State Key Laboratory of Microbial Resources, Institute of Microbiology, Chinese Academy of Sciences, Beijing, China

2 College of Life Sciences, University of Chinese Academy of Sciences, Beijing, China

3 College of Life Sciences, Sichuan Normal University, Sichuan, China

*Correspondence: Hua Xiang, xiangh@im.ac.cn; Dahe Zhao, zhaodh@im.ac.cn

Postal address: State Key Laboratory of Microbial Resources, Institute of Microbiology, Chinese Academy of Sciences, NO.1 Beichen West Road, Chaoyang District, Beijing, 100101, China

Telephone/fax number: +86-10-64807472

Contents

1. Classification of *Ca*. Ordosarchaeia

2. Supplementary results on other features of the gene content of *Ca*. Ordosarchaeia

3. List of supplementary figures (in this file)

Fig. S1 Relative abundance and hypersaline adaptation strategy of *Ca*. Ordosarchaeia in soda-saline lake or enrichment samples.

Fig. S2 Phylogenetic analyses of the classes in the phylum *Halobacteriota* with different trimming methods.

Fig. S3 Bayes tree reconstruction of the classes in the phylum *Halobacteriota*.

Fig. S4 Phylogenetic analyses of the classes in the phylum *Halobacteriota* with the removals of the aspartate and glutamate residues.

Fig. S5 Metabolism potentials of *Ca*. Ordosarchaeia and reference lineage.

Fig. S6 Species tree of ALE analysis with node numbers shown.

Fig. S7 Phylogenetic analyses of McrBG subunits.

Fig. S8 Gene context and phylogenetic analyses of HdrDE subunits.

4. List of supplementary tables (S1–S8 in separate excel files; S9 in this file)

Table S1 Statistic and genomic summary of *Ca*. Ordosarchaeia and related lineages in the phylum *Halobacteriota*

Table S2 Reference genomes for phylogenetic and comparative genomic analyses

Table S3 Average amino acid identity and 16S rRNA gene identity analyses among *Ca*. Ordosarchaeia and related lineages

Table S4 Statistic summary of *Ca*. Ordosarchaeia-related samples

Table S5 Functional assignment of the encoding genes of five *Ca*. Ordosarchaeia genomes using arCOG and eggNOG

Table S6 Functional genes of *Ca*. Ordosarchaeia and reference lineages as well as ancestral and evolutionary inference

Table S7 Summary of methanogenesis-related marker proteins in archaea

Table S8 Genes of interest as well as up- and down-stream genes in the genomes of representative members

Table S9 *Halobacteria* taxa that maintain the cells intact under nonsaline conditions

Classification of *Candidatus* Ordosarchaeia

Description of the class *Candidatus* Ordosarchaeia and taxa classified in the class

Description of *Ca*. Ordosarchaeia class nov. (Or.dos.ar.chae'ia. N.L. neut. n. *Ordosarchaeum*, a candidate genus; N.L. neut. pl. n. suff. *-ia*, ending to denote a class; N.L. neut. pl. n. *Ordosarchaeia*, the *Ordosarchaeum* class). The type order of the class is *Candidatus* Ordosarchaeales.

Description of *Ca*. Ordosarchaeales ord. nov. (Or.dos.ar.chae.a'les. N.L. neut. n. *Ordosarchaeum*, a candidate genus; L. fem. pl. n. suff. *-ales*, ending to denote an order; N.L. fem. pl. n. *Ordosarchaeales*, the *Ordosarchaeum* order). The type family of the order is *Candidatus* Ordosarchaeaceae.

Description of *Ca*. Ordosarchaeaceae fam. nov. (Or.dos.ar.chae.a.ce'ae. N.L. neut. n. *Ordosarchaeum*, a candidate genus; L. fem. pl. n. suff. *-aceae*, ending to denote a family; N.L. fem. pl. n. *Ordosarchaeaceae*, the *Ordosarchaeum* family). The type genus of the family is *Candidatus Ordosarchaeum*.

Description of *Ca*. Ordosarchaeum gen. nov. (Or.dos.ar.chae'um. N.L. n. *Ordos*, Ordos Plateau in Inner Mongolia, China; N.L. neut. n. *archaeum*, archaeon; from Gr. masc. adj. *archaîos*, ancient; N.L. neut. n. *Ordosarchaeum*, a candidate genus). The type species of the genus is *Candidatus* Ordosarchaeum halalkaliphilum.

Description of *Ca*. Ordosarchaeum halalkaliphilum sp. nov. (hal.al.ka.li'phi.lum. Gr. masc. n. *hals (gen. halos)*, salt; N.L. neut. n. *alkali*, alkali; N.L. adj. *philus -a -um*, friend, loving; from Gr. adj. *philos -ê -on*, loving; N.L. neut. adj. *halalkaliphilum*, salt and alkali-loving). The type material is the metagenome-assembled genome Ods01, whose GenBank accession number (WGS) is JAQZCZ000000000.

Supplementary results on other features of the gene content of *Ca.* Ordosarchaeia

In the five representative MAGs of *Ca*. Ordosarchaeia obtained in the research, most of the genes of the archaeal DNA replication complex were annotated (Table S6). In addition, the encoding gene of the bacterial DNA primase DnaG was found. Four genomes contain the genes of Cdc6-related protein, which participates in the replication initiation of chromosomes and megaplasmids.

The members of *Ca*. Ordosarchaeia harbor the encoding genes of diverse DNA repair systems (Table S6). Homologous recombination (single-stranded DNA-specific exonuclease RecJ, recombinase RecA/RadA, and archaeal type of Holliday junction resolvase) and base excision repair (DNA glycosylases MutY and AlkA) might play a role considering the presence of the relevant marker genes. The encoding gene of deoxyribodipyrimidine photolyase repairing ultraviolet damage in DNA was predicted. However, the encoding genes of MutSLH, UvrABC, and DNA end-binding protein Ku functioning in mismatch repair, nucleotide excision repair, and non-homologous end-joining, respectively, were not found. In fact, the *uvrABC* genes of nucleotide excision repair are also absent in *Methanonatronarchaeia*, whereas they are widely distributed in *Halobacteria*, *Ca*. Hikarchaeia, *Methanomicrobia*, and some other classes.

The genomes of Ods04 and Ods05 contain the sequences of ssDNA and dsDNA phages (Table S5). Correspondingly, the two genomes lack a restriction–modification system and CRISPR-Cas system. Ods04 harbors the encoding gene of SoFic protein, which is involved in posttranslational AMPylation (i.e., AMPylase). In fact, SoFic is involved in diverse molecular activities and biological functions. The physiological function of SoFic in Ods04 remains unclear.

The genome of Ods01 harbors the type II restriction–modification system, whereas Ods02 has diverse defense systems including the type I and II restriction–modification systems and the subtype I-B CRISPR-Cas system. The defense systems might protect the hosts from infection by phages.

The sparse existence of the encoding genes of transposases in the genomes of *Ca*. Ordosarchaeia species indicates the possibility of the presence of inserted sequences (Table S6).

Supplementary Figures

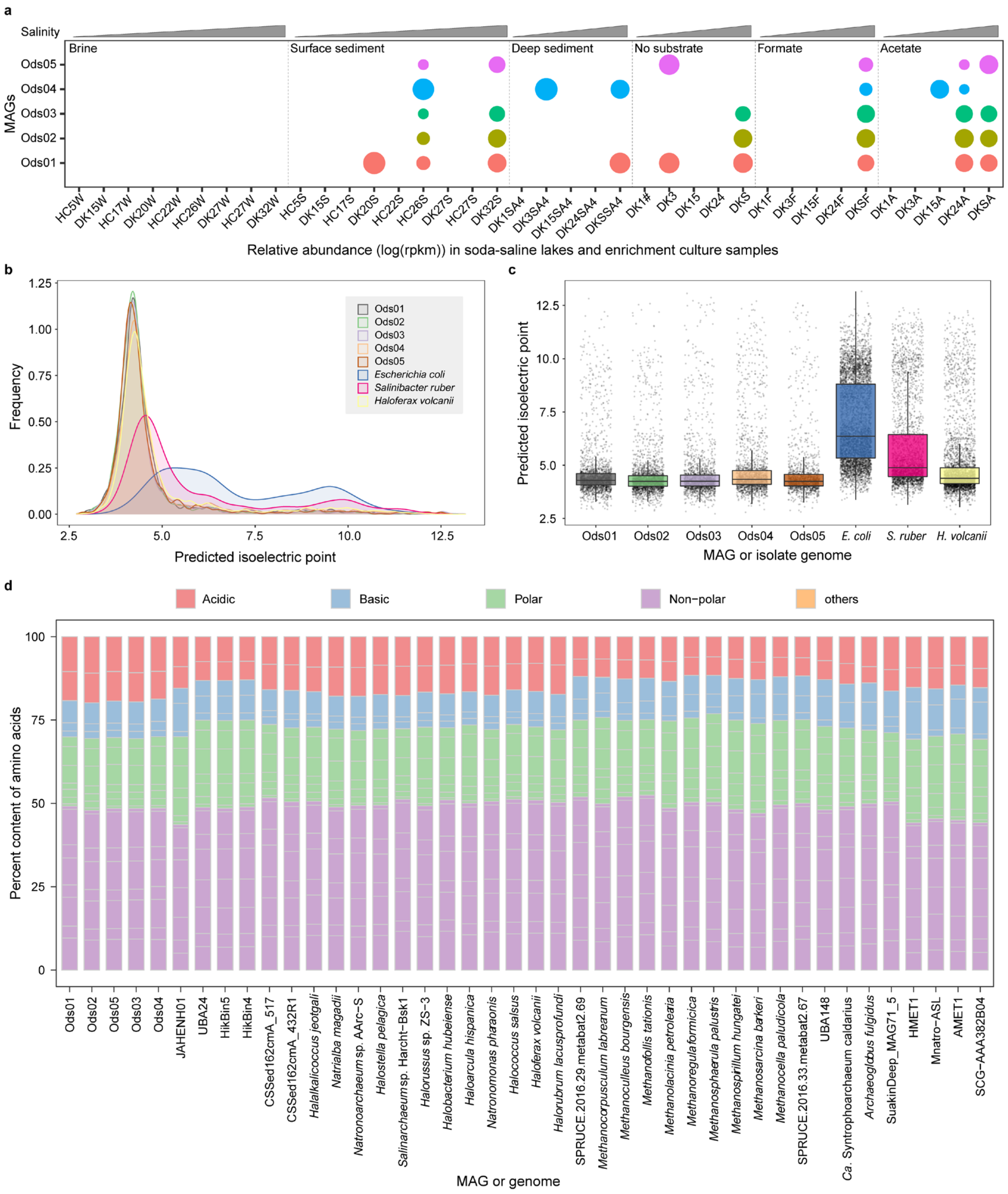

Fig. S1 Relative abundance and hypersaline adaptation strategy of *Ca*. Ordosarchaeia in soda-saline lakes or enrichment samples.

a. Relative abundance of obtained *Ca*. Ordosarchaeia is expressed as rpkm in soda-saline lakes (brine, surface sediment, and deep sediment samples) and the enrichment cultures of deep sediments with different conditions (no substrate, formate, and acetate added). Dot size presents the logarithm of the rpkm. The metagenomic sequencing data of samples could be found via the BioProject identifiers provided in the Data

Availability. The different salinities of the samples are illustrated. # No enough DNA from the sample DK1 for metagenomic sequencing.

b. Isoelectric point profiles of the predicted proteomes. The plots show the kernel density estimate with bin width 0.1 of isoelectric points in the proteomes. c. The box plot displays the distribution of the isoelectric points. It conventionally visualizes the median, the first and third quartiles (lower and upper hinges), and extended 1.5x IQR (inter-quartile range, or distance between the first and third quartiles; shown by lower and upper whiskers). GenBank assembly accession numbers of reference genomes: *Escherichia coli* O157:H7 str. Sakai (GCA_000008865.2), *Salinibacter ruber* DSM 13855 (GCA_000013045.1), and *Haloferax volcanii* DS2 (GCA_000025685.1).

d. Composition of amino acids calculated from the predicted proteomes based on the genome sequences (listed in Table S1).

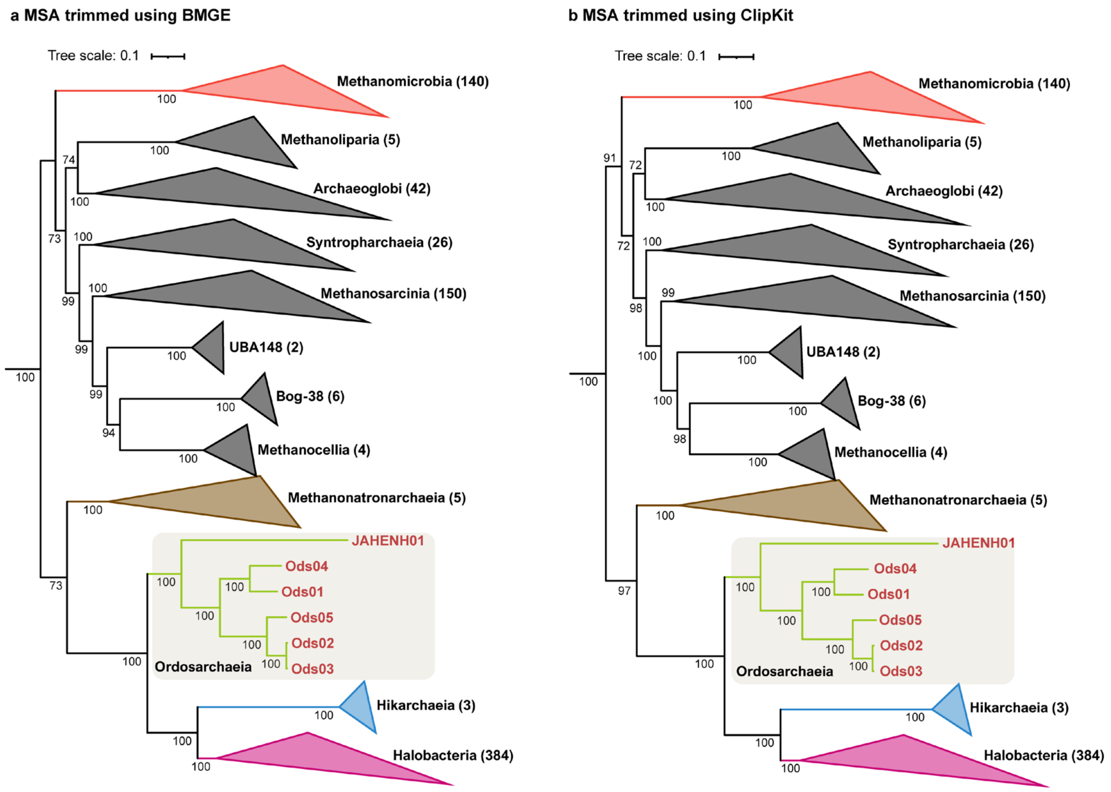

Fig. S2 Phylogenetic analyses of the classes in the phylum *Halobacteriota* with different trimming methods.

The multiple sequence alignment (MSA) of the 53 archaeal marker proteins prior to maximum-likelihood tree reconstruction (also the LG+F+G4 model) was trimmed using BMGE (a) and ClipKIT (b) for the removal of phylogenetically uninformative fast-evolving sites. The number at the node represents the percentage of >70% ultrafast bootstrap support after 1,000 iterations. Some branches are collapsed, and the number in the bracket represents the number of sequences. Both trees reveal the same topology at the class level as that based on the untrimmed MSA in Fig. 2a.

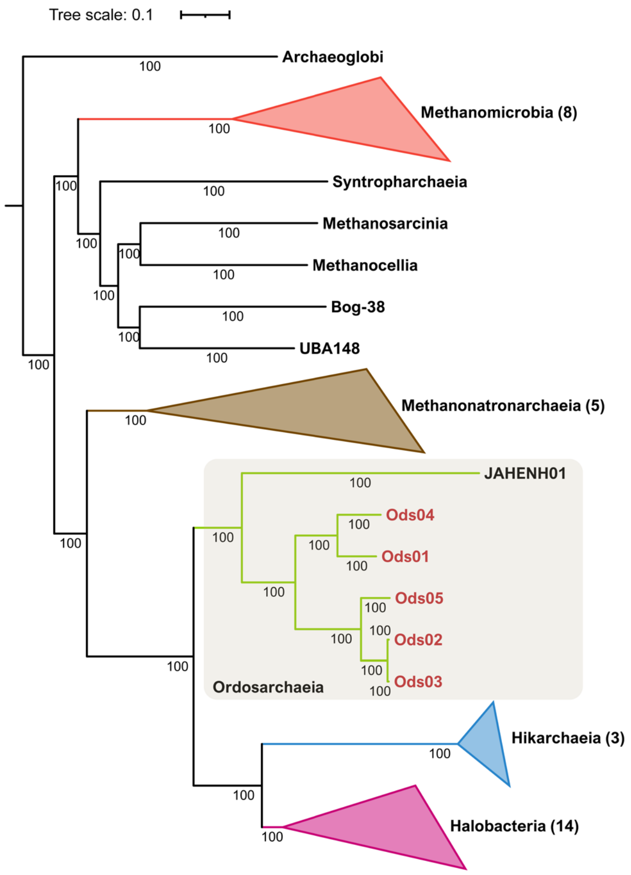

Fig. S3 Bayes tree reconstruction of the classes in the phylum *Halobacteriota*. The phylogenomic trees are based on the 53 archaeal marker proteins, and the reference members are listed in Table S2. The detailed parameters are described in the Methods. The number at the node represents the percentage of the posterior probability of each partition. Some branches are collapsed, and the number in the bracket represents the number of genomes.

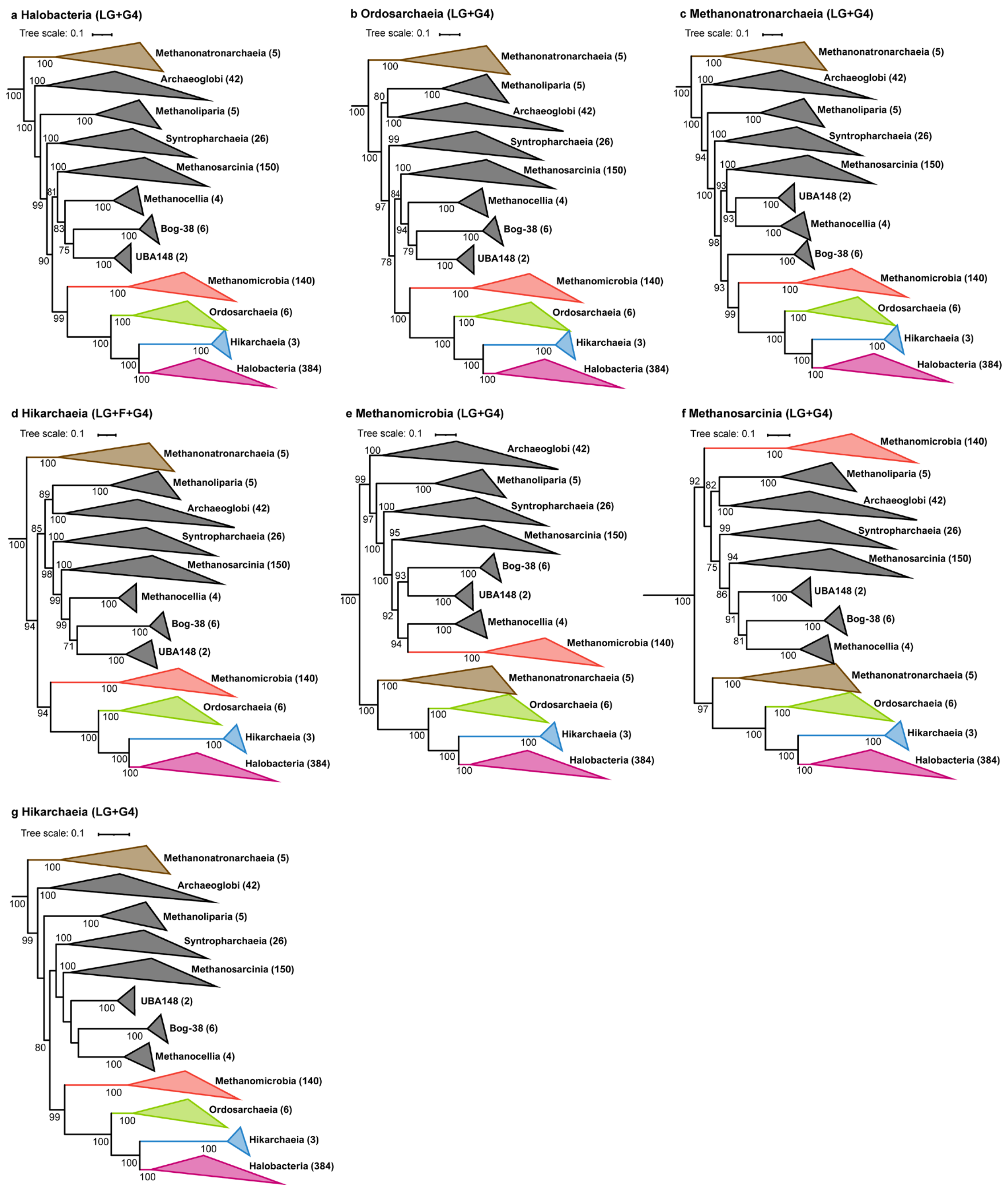

Fig. S4 Phylogenetic analyses of the classes in the phylum *Halobacteriota* with the removals of the aspartate and glutamate residues.

The MSA of the 53 archaeal marker proteins was the same as that in Fig. 2a but the aspartate and glutamate residues were removed by referencing the locations in different lineages: *Halobacterium salinarum* (GCF_008124605.1) of *Halobacteria* (a), Ods02 (JAQZDA000000000) of *Ca*. Ordosarchaeia (b), *Candidatus* Methanohalarchaeum thermophilum (GCA_001914405.1) of *Methanonatronarchaeia* (c), UBA24 (GCA_002503845.1) of *Ca*. Hikarchaeia (d, g), *Methanolacinia petrolearia*

(GCF_000147875.1) of *Methanomicrobia* (e), and *Methanosarcina barkeri* (GCF_000970025.1) of *Methanosarcinia* (f). All the maximum-likelihood trees (a-c, e, f) were reconstructed using the LG+G4 model chosen according to ModelFinder except the LG+F+G4 model in *Ca*. Hikarchaeia (d); therefore, the LG+G4 model was specified (g). The number at the node represents the percentage of ultrafast bootstrap support (>70%) after 1,000 iterations. Some branches are collapsed, and the number in the bracket represents the number of sequences.

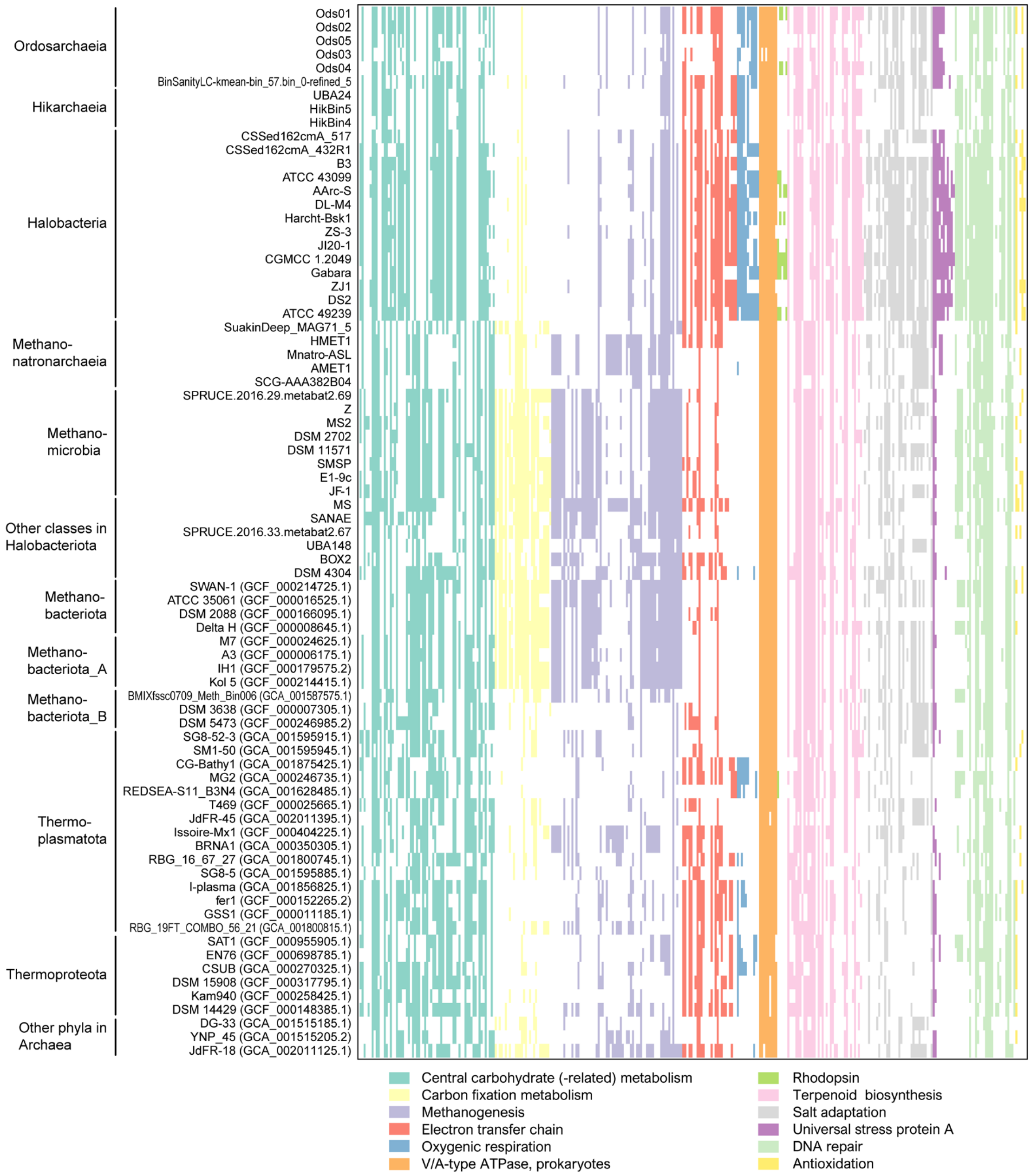

Fig. S5 Metabolism potentials of *Ca*. Ordosarchaeia and reference lineage.

The colored line displays the presence of functional genes in the genomes. The order of the genes from left to right and their functions are listed in Table S6a. The accession numbers of the genomes in the phylum *Halobacteriota* are listed in Table S1.

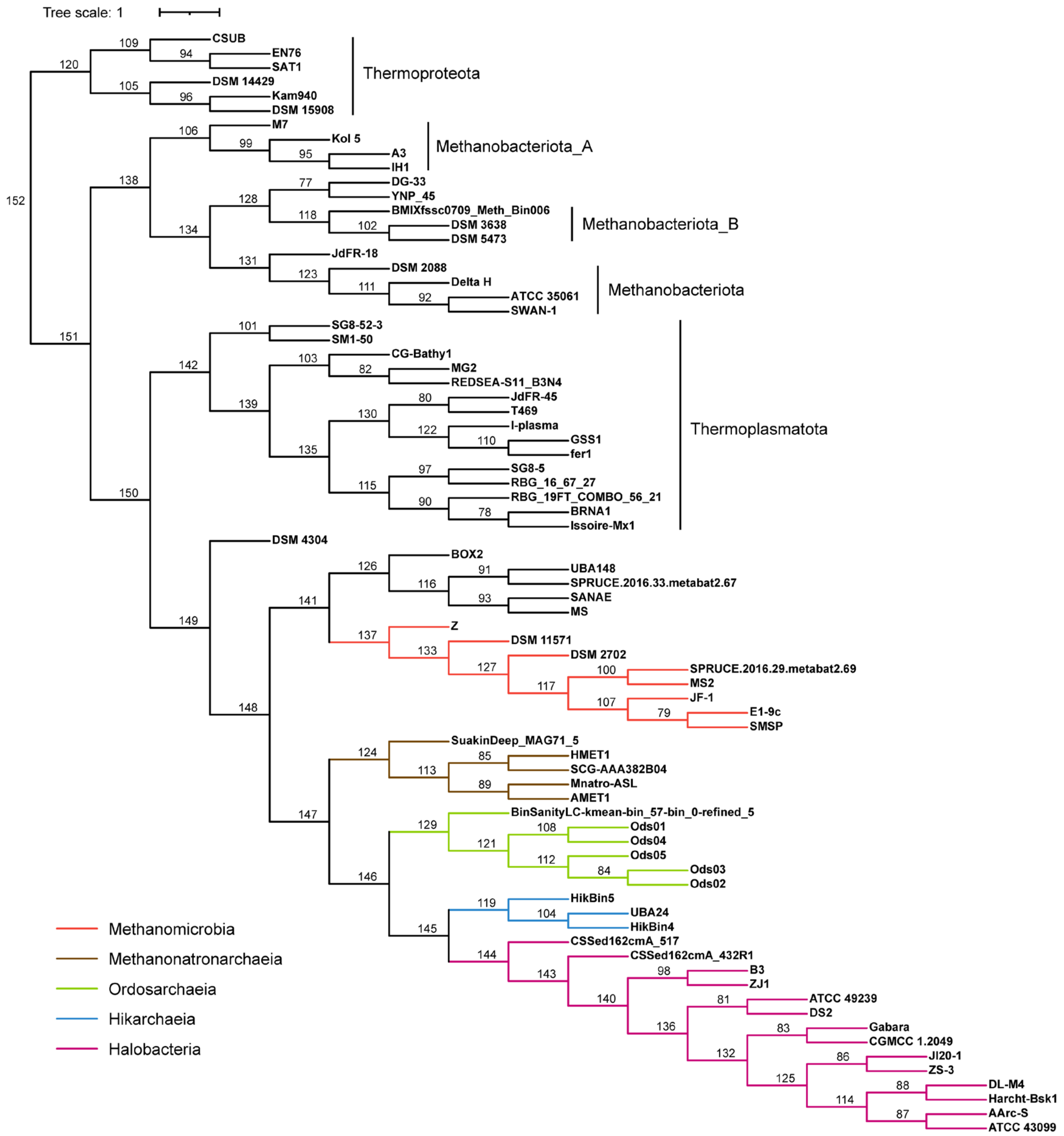

Fig. S6 Species tree of ALE analysis with node numbers shown.

The species tree was extracted from the ALE analyses to show node numbers. A maximum-likelihood tree was read as input for the ALE analysis. During the ALE analysis, only the topology was used and the branch lengths were not used.

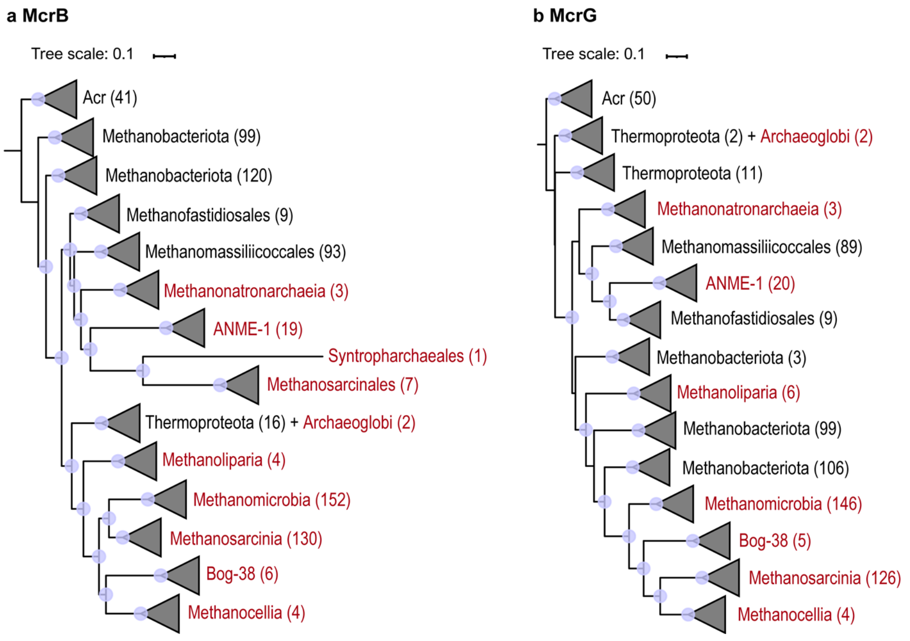

Fig. S7 Phylogenetic analyses of McrBG subunits.

Two trees show different subunits: a. McrB and homolog (LG+F+I+G4 model); b. McrG and homolog (LG+I+G4 model). The circle at the node signifies an ultrafast bootstrap support of >70% based on 1 000 iterations. Some branches are collapsed, and the number in the bracket represents the number of sequences. The red label indicates that the sequences were affiliated with lineages of the phylum *Halobacteriota*. All the sequences are listed in Table S7. The detailed approaches are described in the Methods.

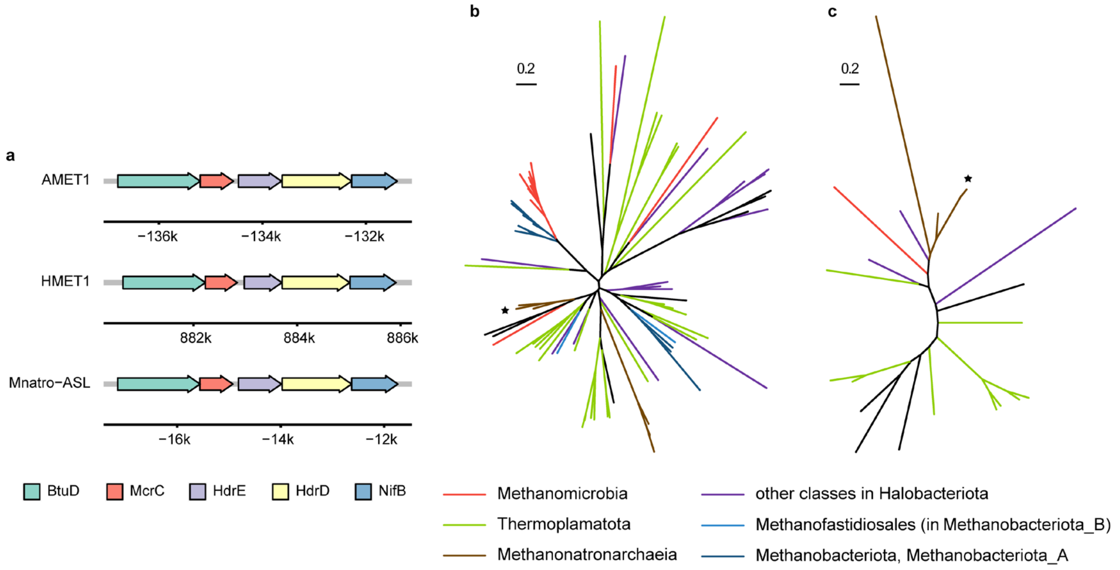

Fig. S8 Gene context and phylogenetic analyses of HdrDE subunits.

a. Arrangement of *hdrDE* genes in *Methanonatronarchaeia* genomes. The contig ID and position of each gene cluster are listed in Table S8. b. Phylogeny of HdrD. c. Phylogeny of HdrE. The colored branches mark the different taxonomic sources of the proteins in the Archaea. The black stars indicate the sequences whose genes are located in the *mcrC-hdrDE* cluster.

Supplementary Table

Table S9 *Halobacteria* taxa that maintain the cells intact under nonsaline conditions

Number	Species	Genus	Reference
1	*Halalkalicoccus tibetensis*	*Halalkalicoccus*	[S1]
2	*Halalkalicoccus jeotgali*	*Halalkalicoccus*	[S2]
3	*Halalkalicoccus paucihalophilus*	*Halalkalicoccus*	[S3]
4	*Halalkalicoccus subterraneus*	*Halalkalicoccus*	[S4]
5	*Haladaptatus cibarius*	*Haladaptatus*	[S5]
6	*Haladaptatus halobius*	*Haladaptatus*	[S6]
7	*Haladaptatus salinisoli*	*Haladaptatus*	[S6]
8	*Haladaptatus litoreus*	*Haladaptatus*	[S7]
9	*Haladaptatus pallidirubidus*	*Haladaptatus*	[S8]
10	*Haladaptatus paucihalophilus*	*Haladaptatus*	[S9]
11	*Halocatena pleomorpha*	*Halocatena*	[S10]
12	*Halocatena salina*	*Halocatena*	[S11]
13	*Halomarina oriensis*	*Halomarina*	[S12]

Supplementary references

S1. Xue Y, Fan H, Ventosa A, Grant WD, Jones BE, Cowan DA, et al. *Halalkalicoccus tibetensis* gen. nov., sp. nov., representing a novel genus of haloalkaliphilic archaea. *Int J Syst Evol Microbiol*. 2005;55(6):2501-5.

S2. Roh SW, Nam YD, Chang HW, Sung Y, Kim KH, Oh HM, et al. *Halalkalicoccus jeotgali* sp. nov., a halophilic archaeon from shrimp jeotgal, a traditional Korean fermented seafood. *Int J Syst Evol Microbiol*. 2007;57(10):2296-8.

S3. Liu BB, Tang SK, Zhang YG, Lu XH, Li L, Cheng J, et al. *Halalkalicoccus paucihalophilus* sp. nov., a halophilic archaeon from Lop Nur region in Xinjiang, northwest of China. *Antonie van Leeuwenhoek*. 2013;103(5):1007-14.

S4. Chen S, Xu Y, Sun S, Chen F, Liu J. *Halalkalicoccus subterraneus* sp. nov., an extremely halophilic archaeon isolated from a subterranean halite deposit. *Antonie van Leeuwenhoek*. 2019;112(7):1067-75.
S5. Roh SW, Lee ML, Bae JW. *Haladaptatus cibarius* sp. nov., an extremely halophilic archaeon from seafood, and emended description of the genus Haladaptatus. *Int J Syst Evol Microbiol*. 2010;60(5):1187-90.
S6. Xin YJ, Bao CX, Tan S, Hou J, Cui HL. *Haladaptatus halobius* sp. nov. and *Haladaptatus salinisoli* sp. nov., two extremely halophilic archaea isolated from Gobi saline soil. *Int J Syst Evol Microbiol*. 2022;72(10):005543.
S7. Cui HL, Sun FF, Gao X, Dong Y, Xu XW, Zhou YG, et al. *Haladaptatus litoreus* sp. nov., an extremely halophilic archaeon from a marine solar saltern, and emended description of the genus Haladaptatus. *Int J Syst Evol Microbiol*. 2010;60(5):1085-9.
S8. Liu BB, Zhao WY, Chu X, Hozzein WN, Prabhu DM, Wadaan MA, et al. *Haladaptatus pallidirubidus* sp. nov., a halophilic archaeon isolated from saline soil samples in Yunnan and Xinjiang, China. *Antonie van Leeuwenhoek*. 2014;106(5):901-10.
S9. Savage KN, Krumholz LR, Oren A, Elshahed MS. *Haladaptatus paucihalophilus* gen. nov., sp. nov., a halophilic archaeon isolated from a low-salt, sulfide-rich spring. *Int J Syst Evol Microbiol*. 2007;57(1):19-24.
S10. Verma A, Pal Y, Kumar P, Krishnamurthi S. *Halocatena pleomorpha* gen. nov. sp. nov., an extremely halophilic archaeon of family Halobacteriaceae isolated from saltpan soil. *Int J Syst Evol Microbiol*. 2020;70(6):3693-700.
S11. Wu Z-P, Zheng X-W, Sun Y-P, Wang B-B, Hou J, Cui H-L. *Halocatena salina* sp. nov., a filamentous halophilic archaeon isolated from Aiding Salt Lake. *Int J Syst Evol Microbiol*. 2022;72(12):005637.
S12. Inoue K, Itoh T, Ohkuma M, Kogure K. *Halomarina oriensis* gen. nov., sp. nov., a halophilic archaeon isolated from a seawater aquarium. *Int J Syst Evol Microbiol*. 2011;61(4):942-6.
